# Characterization and Corrosion Resistance of Boron-Containing-Austenitic Stainless Steels Produced by Rapid Solidification Techniques

**DOI:** 10.3390/ma11112189

**Published:** 2018-11-05

**Authors:** Guilherme Y. Koga, Lucas B. Otani, Ana M. B. Silva, Virginie Roche, Ricardo P. Nogueira, Alberto M. Jorge, Claudemiro Bolfarini, Claudio S. Kiminami, Walter J. Botta

**Affiliations:** 1 Department of Materials Science and Engineering, Federal University of São Carlos, Rod. Washington Luis, km 235, CEP 13565-905 São Carlos, SP, Brazil; lucasbotani@gmail.com (L.B.O.); aninha_vilasboas@yahoo.com.br (A.M.B.S.); moreira@ufscar.br (A.M.J.J.); cbolfa@ufscar.br (C.B.); kiminami@ufscar.br (C.S.K.); wjbotta@ufscar.br (W.J.B.); 2 LEPMI, UMR5279 CNRS, Grenoble INP, Université Grenoble Alpes, 1130, rue de la piscine, BP 75, 38402 Saint Martin d’Hères, France; virginie.roche@lepmi.grenoble-inp.fr (V.R.); ricardo.nogueira@ku.ac.ae (R.P.N.); 3 Gas Research Center, Khalifa University of Science and Technology, P.O. Box 2533, Abu Dhabi, UAE

**Keywords:** rapid solidification, stainless steel, bulk amorphous alloys, thermo-calc, corrosion

## Abstract

The composition of a commercial duplex stainless steel was modified with boron additions (3.5, 4.5, and 5.5 wt.%) and processed by rapid-quenching techniques: Melt-spinning, copper-mold casting, and high-velocity oxygen fuel (HVOF). Spray deposition was also used to produce alloys as the process may induce rapid-solidified-like microstructures. These processing routes led to microstructures with distinguished corrosion resistance. Among the alloys with different boron contents, the 63.5Fe25Cr7Ni4.5B composition enabled the production of fully amorphous ribbons by melt-spinning. The cooling rate experienced during copper-mold casting, high-velocity oxygen fuel, and spray deposition did not ensure complete amorphization. The crystalline phases thereby formed were (Fe,Cr)_2_B and (Fe,Mo)_3_B_2_ borides in an austenitic-matrix with morphology and refinement dependent of the cooling rates. Fully amorphous 63.5Fe25Cr7Ni4.5B ribbons exhibited outstanding corrosion resistance in chloride-rich alkaline and acid media with negligible corrosion current densities of about 10^−8^ A/cm² and a broad passivation plateau. Although the specimens of the same composition produced by HVOF process and spray deposition exhibited lower corrosion resistance because of intrinsic porosity and crystalline phases, their corrosion behaviors were superior to those of AISI 1045 steel used as substrate with the advantage to be reinforced with hard borides known to be resistant against wear.

## 1. Introduction

Rapid solidification processed alloys have been attracting scientific and industrial attention over the last decades, and they have opened new possibilities in metallurgy fields regarding the design of microstructures with improved physical and mechanical properties [1]. For instance, bulk amorphous steels (BAS) have been a subject of great interest due to their unique characteristics such as excellent magnetic properties, high hardness and fracture strength, and an attractive combination of resistance to corrosion and wear [2,3,4,5]. Compared to other bulk amorphous alloy systems (e.g., Pd- and Zr-based alloys), BAS have much lower material cost and higher thermal stability which are attractive for industrial applications [6,7,8,9]. However, the glass forming ability (GFA) of the designed BAS has to be sufficiently high to ensure amorphization by using commercial-grade raw materials, such as steels and iron-alloys.

The addition of minor relatively low-cost alloying elements to cast-iron and steels has been reported to be effective for amorphous phase formation in melt-spun ribbons and cast cylinders [10,11]. For instance, Inoue and Wang [12] have reported that the addition of a small amount of B (0.4 wt.%) to the commercial FC20 cast iron allows the formation of an amorphous phase in as-spun ribbons and copper cast samples. Similarly, Cheney and Vecchio [13] have confirmed the high GFA of the boron and niobium modified AISI 430 stainless steel, which exhibited a broad supercooled liquid region (SLR) with a ΔTx of 67 K. However, the small critical thickness or diameter sections (dimensions ranging from 10^−3^ to 10^−2^ m) and the poor room temperature plasticity significantly restrict their applicability as structural materials [14]. In such way, to solve the inherent issue of dimensional limitations of Fe-based amorphous alloys, the coating technology is being considered attractive to benefit from their excellent resistance to wear and corrosion. Therefore, amorphous high wear and corrosion resistant coatings have been produced by thermal spraying processes [15,16,17]. Even though spray forming is not considered a rapid solidification technique, the microstructure evolution of materials processed by this route exhibits interesting features resulting from two completely different stages of solidification [18]: At the atomization (high cooling rates, ranging from 10^2^ to 10^4^ K/s) and after deposition (low cooling rates, ranging from 0.1 to 10 K/s). Regardless of the second solidification stage, it is reported that Fe-based deposits contain an amorphous phase in the microstructure [19,20]. Besides the amorphous phase formation in some alloys, a characteristic advantage of applying this technique is the features of the final microstructure, which presents low levels of segregation from an alloy coming directly from the liquid. Concerning the corrosion resistance, the design of BAS through stainless steel modification is more interesting than the use of mild steel or cast iron as master alloys [21]. Compared to crystalline steels, amorphous ones have high surface energy which means that the dissolution of low corrosion resistant elements in aqueous media is high. It implies that amorphous alloys steel with no addition of corrosion resistant elements such as Cr, Nb or Mo are more prone to corrode than their crystalline counterparts [22]. In contrast, the presence of Cr as low as 4–6 at.% in amorphous steels enables the formation of a highly stable and resistant passivating film because of the fast and selective dissolution of Fe and consequent efficient enrichment of corrosion resistant elements on the surface [23]. In this case, the ultimate corrosion resistance of Cr-containing BAS is superior to their crystalline counterparts. Moreover, alloys that withstand extreme corrosion conditions can be designed since Fe-based amorphous alloys also allow the formation of superior Cr solid solution than crystalline equivalents; therefore, enabling notable improvement of corrosion resistance [22]. With respect to the boron addition, the increase of B content not only improves the GFA of Fe-Cr-B-based alloys but also is reported to promote superior corrosion resistance in acid media [24].

Besides the interest in the BAS, other microstructures resulting from rapid solidification of Fe-based alloys have been proved to present interesting characteristics, such as the extension of the solid solubility, enhanced compositional flexibility, and formation of refined crystalline microstructure with reduced segregation levels [25]. For instance, even in cases where amorphization is not fully achieved in boron modified stainless steels, a refined stainless steel microstructure reinforced with hard intermetallic phases with an interesting combination of corrosion and wear resistance can be produced [26,27,28].

To date, few studies focusing on the production and corrosion investigation of rapid solidified commercial stainless steels modified with different boron addition are available, most of them being related to the modified ferritic and martensitic stainless steels. In this work, the 68-xFe25Cr7NixB austenite stainless steels with different B contents (x = 3.5, 4.5, and 5.5 wt.%) were manufactured. The samples were obtained through different rapid-quenching routes: Melt-spinning, copper-mold casting, and high-velocity oxygen fuel (HVOF). Spray deposition was also used because of the possibility to induce rapid-solidified-like microstructures. Corrosion measurements were carried out in alkaline and acid chloride-rich media to evaluate the corrosion resistance of the alloys, coatings, and deposits. Results point out a strong corrosion resistance dependency regarding the resulted microstructure induced by processes with different cooling rates.

## 2. Experimental Procedure

### 2.1. Amorphization Study

The 68-xFe25Cr7NixB (x = 3.5, 4.5, and 5.5 wt.%) alloys were selected for this study to evaluate the impact of boron addition, from a commercial iron-boron alloy (with 16.5 wt.% B, 0.3 wt.% C, and 0.57 wt.% Si), on the amorphization of a commercial duplex stainless steel (SAF 2205). The nominal compositions were adjusted by adding pure chromium and nickel, Table 1, to ensure high corrosion resistance.

Melt-spinning technique and suction copper-mold casting were used to investigate the GFA of the alloys. Ingots of 15 g used in each process were previously prepared under a purified Ti-gettered argon atmosphere arc melter (model AM, Edmund Bühler GmbH, Bodelshausen, Germany). The ingots were re-melted several times to ensure compositional homogeneity. The pre-alloyed ingot was induction-re-melted in a quartz tube and ejected on a copper wheel rotating at a speed of 50 m/s in an argon atmosphere to produce ribbons. Cylindrically shaped samples with length of about 10 mm and different diameters (Ø) in the range of 2 to 12 mm were produced by sucking the molten alloy into a large copper mold due to the difference in gas pressure of 20 kPa.

### 2.2. High-Velocity Oxygen Fuel (HVOF) Process

Based on the results of Section 2.1, the 63.5Fe25Cr7Ni4.5B composition was selected to produce coatings because of its higher GFA among the 68-xFe25Cr7NixB (x = 3.5, 4.5, and 5.5 wt.%) alloys. Feedstock powders were produced by gas-atomization to be used in the HVOF process to produce coatings. The 63.5Fe25Cr7Ni4.5B alloy was melted in argon atmosphere and sprayed using nitrogen (gas-flow-rate of 3.8 m^3^/min), mass-flow-rate of 5.3 kg/min, temperature of 1600 °C, and nozzle of Ø 6 mm. After sieving, particles with size inferior to 45 µm were selected as feedstock powder. HVOF coatings were sprayed onto sandblasted and degreased mild steel (AISI 1045) 100 mm × 30 mm × 5 mm plates, with a standoff distance of 350 mm, and flame temperature of about 2900 °C. A spray system TAFA JP-5000 HP/HVOF (Praxair Surface Technologies, Indianapolis, IN, USA) and a 5220-model gun were used to produce the coatings.

### 2.3. Spray Forming

The same composition with the higher GFA (63.5Fe25Cr7Ni4.5B) was processed by spray forming to evaluate the resulting microstructure and its impact on the corrosion resistance. Mild steel (AISI 1045) discs measuring Ø 47 mm × 5 mm were used as a substrate to produce deposits by spray deposition. The alloy was molten under argon atmosphere and sprayed onto the substrate using nitrogen gas (gas-to-flow rate of 8.7 m^3^/min), mass-to-flow rate of 14.0 kg/min, flying distance of 500 mm, nozzle of Ø 6 mm, and temperature of 1600 °C.

### 2.4. Characterization Techniques

The samples were characterized by scanning electron microscopy (SEM), in a Philips (FEI, Hillsboro, OR, USA) XL30 FEG equipped with energy dispersive spectroscopy (EDS) (OXFORD–LINK ISIS 300, High Wycombe, UK), and the phase constituents identified using X-ray diffraction (XRD) analysis performed on an X-ray diffractometer Rigaku Geigerflex ME210GF2 (Tokyo, Japan), with Cu-Kα radiation. The thermal stability was examined by differential scanning calorimeter (DSC), in a Netzsch 404 (Selb, Germany), at the heating rate of 0.67 K/s. Transmission electron microscopy (TEM) in a Philips (FEI, Hillsboro, OR, USA) CM 120 operated at 120 kV was carried out in the 63.5Fe25Cr7Ni4.5B as-spun ribbons. To better address the microstructure evolution and to evaluate how the boron additions may influence the phase stability, thermodynamic calculations were performed using Thermo-Calc^®^ software [29] version 4.0 equipped with TCFE7 database considering equilibrium conditions. The information obtained through these predictions, phase formation sequence and the amount of phase, were essential to indicate which phases, among many possibilities, are most likely to appear.

### 2.5. Electrochemical Measurements

Polarization curves were carried out using a conventional three electrodes cell set-up at room temperature, 25 °C, in a potentiostat model 1287 SOLARTRON (Farnborough, UK) and software CORRWARE 2. The working electrodes (WE) with 1 cm² area were the 63.5Fe25Cr7Ni4.5B samples processed by melt-spinning, HVOF, and spray forming. For comparison reasons, the AISI 1045 steel substrate for HVOF and spray deposition and the SAF 2205 base alloy were also used as WE. The counter electrode was a platinum (Pt) sheet, and a saturated calomel electrode (SCE) was used as the reference electrode. Electrochemical measurements were performed in chloride-rich acid (pH = 3.0), neutral (pH = 5.5), and alkaline (pH = 10.0) solutions containing 35 g/L of NaCl, typical of marine environments. In this case, the solutions were prepared by using deionized water and additions of H_2_SO_4_ and NaOH to adjust the pH of acid and alkaline electrolytes, respectively. Before the analysis, the solutions were subjected to air bubbling for 30 min to assure oxygen saturation. Potentiodynamic polarizations, with a scan rate of 1 mV/s, were launched after 30 min of immersion of the sample in the solution to allow steady free potential conditions to be reached. Corrosion current density (*i_corr_*) and the corrosion potential (*E_corr_*) were used to evaluate the corrosion behavior of the as-spun ribbons, HVOF coatings, and sprayed deposits. The *i_corr_* was determined by extrapolating the anodic and cathodic Tafel regions around *E_corr_*. Reproducibility of data was verified by repeating the test three times.

## 3. Results and Discussion

### 3.1. Amorphization Study of the 68-xFe25Cr7NixB (x = 3.5, 4.5, and 5.5 wt.%) Alloy Compositions

**Figure 1:**
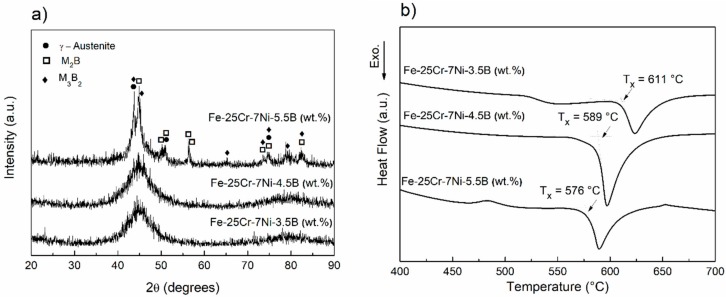
X-ray diffraction (XRD) (**a**) and differential scanning calorimeter (DSC) (**b**) curves of 68-xFe25Cr7NixB (x = 3.5, 4.5, and 5.5 wt.%) as-spun ribbons obtained by melt-spinning.

Figure 1 shows the XRD and DSC curves of the as-spun 68-xFe25Cr7NixB (x = 3.5, 4.5, and 5.5 wt.%) ribbons. A single broad halo peak around 2θ = 45 degrees and no diffraction peaks due to crystalline phases are seen for 64.5Fe25Cr7Ni3.5B and 63.5Fe25Cr7Ni4.5B as-spun ribbons, which indicate high amorphous content (Figure 1a). However, a drastic change in the X-ray diffraction pattern is observed for 5.5 wt.% B. This higher boron addition generated an amorphous phase together with several crystalline phases as indexed in Figure 1a. DSC curves (Figure 1b) present a single exothermic peak, indicating that the amorphous phase of the 68-xFe25Cr7NixB (x = 3.5, 4.5, and 5.5 wt.%) ribbons crystallize in one stage. Moreover, the onset of the crystallization peak increases gradually (from 576 °C to 611 °C) with decreasing the B content. Similar tendencies were reported by Inoue and Wang [12] and Yao et al. [30]. Even if no glass transition temperature (T_g_) was detected for the DSC conditions of the experiment, the non-crystalline solid thereby formed is a metallic glass. Based on Gupta’s [31] definition, glass structure is achieved if the short-range order (SRO) of the non-crystalline solid is equal to the SRO of the melt, which is always obtained by melt-cooling since the structure of the melt is frozen-in during liquid to metallic glass transition. Concerning the amorphous content, the DSC curves corroborate the results observed in the XRD patterns, i.e., the 62.5Fe25Cr7Ni5.5B ribbons exhibit a smaller exothermic crystallization peak compared to the 68-xFe25Cr7NixB (x = 3.5 and 4.5 wt.%) ones. It can be also seen in the DSC curves that 63.5Fe25Cr7Ni4.5B ribbons show a larger crystallization peak than 64.5Fe25Cr7Ni3.5B, suggesting superior GFA among the 68-xFe25Cr7NixB (x = 3.5, 4.5, and 5.5 wt.%) alloy compositions.

**Figure 2:**
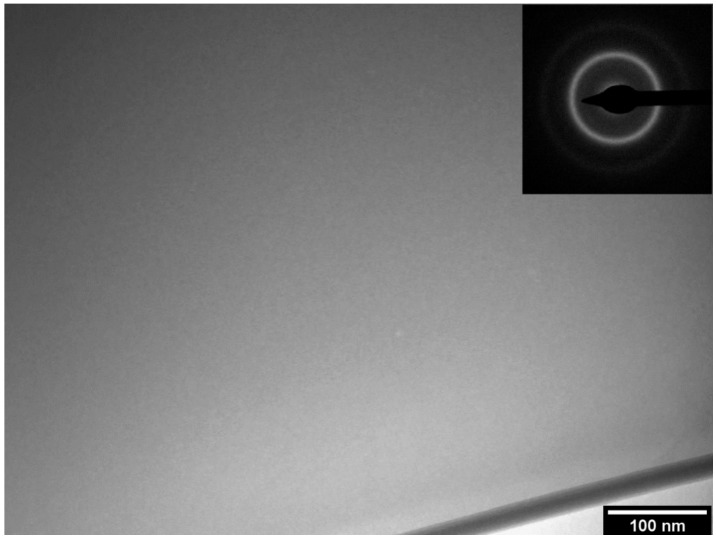
Bright field transmission electron microscopy (TEM) micrograph of the 63.5Fe25Cr7Ni4.5B ribbon obtained by melt-spinning and the selected electron diffraction pattern.

The complete amorphization of the as-spun ribbons of the 63.5Fe25Cr7Ni4.5B alloy was confirmed by TEM, as it can be observed in the bright-field (BF) transmission electron micrograph and its corresponding selected area electron diffraction pattern (SADP) shown in Figure 2. The formation of the amorphous structure was verified by the featureless homogeneous image with no visible difference in contrast, which would characterize any crystalline phase. Additionally, the SADP exhibits only diffuse rings which are typical of fully amorphous structures. Thus, the amorphous phase content of the ribbons with different boron content can be estimated by the V_f_ = ΔH_sample_/ΔH_amorphous_ equation, where ΔH_sample_ is the crystallization enthalpy of the 68-xFe25Cr7NixB (x = 3.5 and 5.5 wt.%) ribbons and ΔH_amorphous_ is the crystallization enthalpy of the fully amorphous 63.5Fe25Cr7Ni4.5B ribbon. Even though the boron contents were slightly different, the obtained DSC curves, Figure 1b, can be used for semi-quantitative calculations of the amorphous content. The estimated amorphous fraction of the 64.5Fe25Cr7Ni3.5B and 62.5Fe25Cr7Ni5.5B ribbons was about 58% and 45%, respectively.

**Figure 3:**
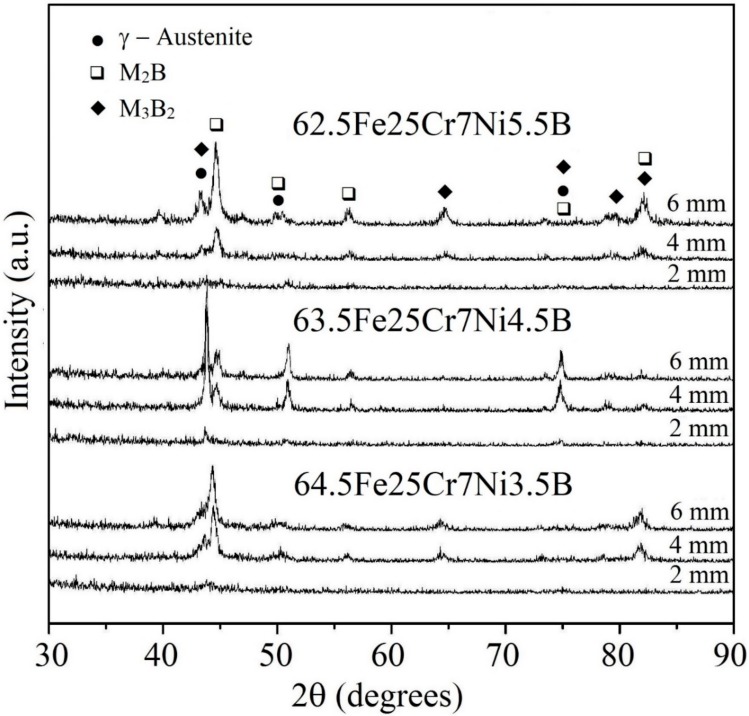
XRD patterns of 68-xFe25Cr7NixB (x = 3.5, 4.5, and 5.5 wt.%) alloy sections extracted from different sample diameters.

**Figure 4:**
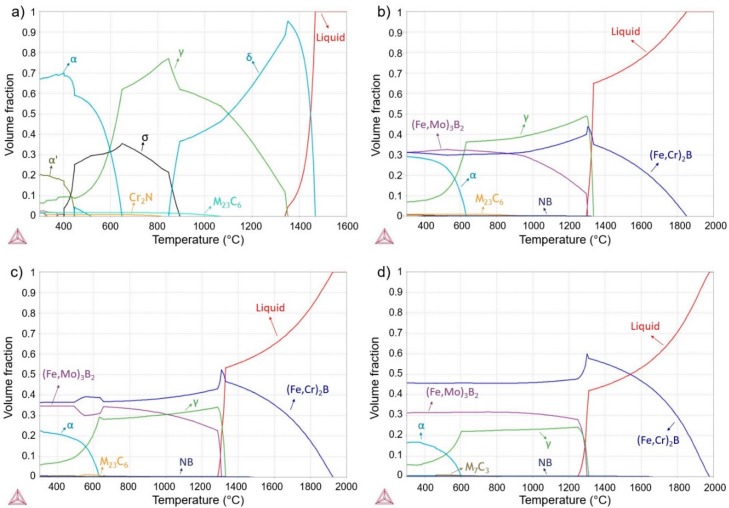
Equilibrium phase evolution with the temperature of the alloy (**a**) without boron addition (i.e., of the SAF superduplex stainless steel); (**b**) with 3.5 wt.% B; (**c**) with 4.5 wt.% B and (**d**) with 5.5 wt.% B.

Further investigation of the amorphization of boron-modified duplex stainless steel was carried out on copper-mold cast samples. Figure 3 shows the XRD patterns of as-cast 68-xFe25Cr7NixB (x = 3.5, 4.5, and 5.5 wt.%) cylinders with different diameters ranging from 2 to 6 mm. All alloy compositions presented an increase of the intensities related to crystalline phases with the increasing of the diameters (Ø). As it can be observed, all the compositions presented crystalline phases when processed by suction cast in copper mold (even for the higher cooling rate associated to the lower cylinder diameter). Thermodynamic simulations were performed, Figure 4, to better understand the microstructure evolution of all compositions upon cooling. Figure 4a depicts phases evolution with the temperature for the SAF 2205 base alloy. From the calculations, this composition is expected to present a duplex temperature (when the volume fraction of both ferrite and austenite are 50 vol.%) with the presence, even if small, of the carbide, nitride and sigma phase in thermodynamic equilibrium with these phases. Figure 4b represents the alloy with 3.5 wt.% B and, from this point, it is possible to observe that the solidification of the alloy is highly influenced by the addition of this element. The boron addition stabilizes the (Fe,Cr)_2_B boride and this phase is primary even for the smaller additions of boron. Due to the chromium retention by the formation of high-temperature borides, the alloy no longer presents a duplex temperature and the austenite is the prevailing phase for a large range of temperatures. Additionally, another boride, the (Fe,Mo)_3_B_2_, is also formed coming from the liquid. This boride is formed in a eutectic manner and, therefore, presents different morphology compared to the (Fe,Cr)_2_B primary boride. Figure 4c,d present the same graphic for 4.5 wt.% B and 5.5 wt.% B, which point out that higher boron contents increase the initial temperature formation of the (Fe,Cr)_2_B phase and, consequently, promotes superior volume fraction of this phase. Increasing the boron content from 3.5 wt.% B to 4.5 wt.% B increased the (Fe,Mo)_3_B_2_ content; however, increasing from 4.5 wt.% B to 5.5 wt.% B decreased the amount of the thermodynamic equilibrium volume fraction of this boride. This result indicates that higher boron content will significantly increase the (Fe,Cr)_2_B stability, but not necessarily the amount of (Fe,Mo)_3_B_2_ boride.

**Figure 5:**
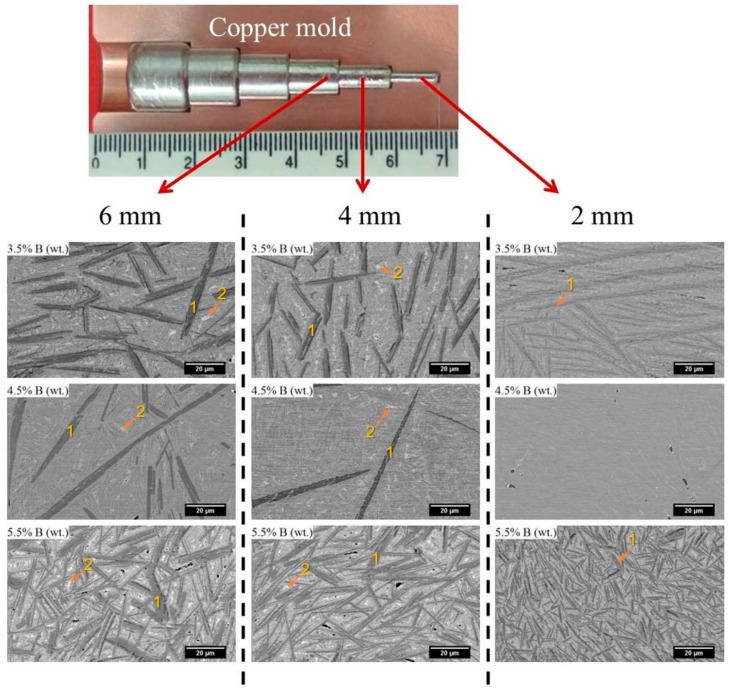
Scanning electron microscopy (SEM) backscattered electron image of 68-xFe25Cr7NixB (x = 3.5, 4.5, and 5.5 wt.%) bulk sections extracted from different diameters of the sample produced by copper-mold casting technique. The ruler is in cm. Regions 1 and 2 correspond to the M_2_B and M_3_B_2_ borides, respectively.

SEM micrographs of different 68-xFe25Cr7NixB (x = 3.5, 4.5, and 5.5 wt.%) sample diameters are shown in Figure 5. The microstructures are composed of bright and dark phases embedded in the austenitic matrix. As described in the equilibrium solidification path preciously presented, upon solidification, the (Fe,Cr)_2_B and (Fe,Mo)_3_B_2_ are formed within the austenitic matrix. The brighter contrast in BSE in Figure 5 suggests the presence of a heavy element compared with the other phases. Indeed, EDS analysis performed on the Ø 6 mm cylinders revealed that the darker phase, region 1, is rich in Cr while the brighter phase, region 2, is rich in Mo. These results point out that the primary phase presented in the microstructure, region 1, is probably the (Fe,Cr)_2_B phase, which is in agreement with the thermodynamic predictions since the primary phase described is the (Fe,Cr)_2_B with the end of the solidification being a eutectic reaction leading to the formation of the (Fe,Mo)_3_B_2_, region 2 in Figure 5. All of these phases were also identified in the XRD patterns, Figure 3, reinforcing the SEM and EDS results. Close to the tip (Ø 2 mm), the samples with 4.5 wt.% B presented the most refined microstructure compared to those with 3.5 and 5.5 wt.% B additions, suggesting that the 63.5Fe25Cr7Ni4.5B alloy is more effective for decreasing the nucleation and growth of crystalline phases. In fact, considering the results presented up to this point, the alloy with higher GFA (4.5 wt.% of B) probably hindered the formation of primary (Fe,Cr)_2_B boride leading to the formation of a lower amount of this phase and almost suppressed it at the lower diameter. Indeed, the microstructures of the 4.5 wt.% B content composition presented a different microstructure compared to the 3.5 and 5.5 wt.% B alloys; both with higher amounts of primary (Fe,Cr)_2_B boride with an eutectic reaction at the end of the solidification, leading to the formation of the (Fe,Mo)_3_B_2_ boride.

### 3.2. Microstructure Characterization of 63.5Fe25Cr7Ni4.5B HVOF Coatings and Spray Formed Deposits

Fully amorphous 63.5Fe25Cr7Ni4.5B as-spun ribbons were obtained by melt-spinning (Figure 1 and Figure 2). However, bulk 63.5Fe25Cr7Ni4.5B copper-mold cast samples were crystalline (Figure 3 and Figure 5). To evaluate the possibility of using this material in a commercially available rapid solidification process, HVOF was chosen to produce thin (about hundreds of micrometers) coatings. The cooling rates that can be achieved in the HVOF are in the order of 10^4^ to 10^6^ K/s, which are high enough to enable many alloy compositions to be deposited above their respective critical cooling rate for amorphization [32]. The 63.5Fe25Cr7Ni4.5B alloy composition was selected for this procedure due to its superior GFA.

**Figure 6:**
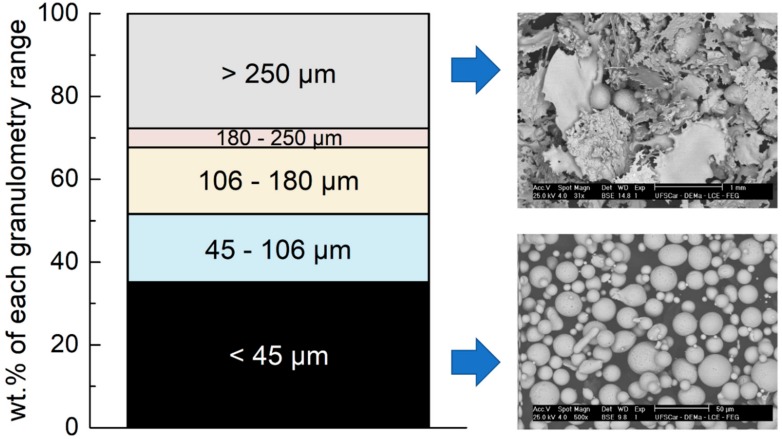
Weight percentage of powders with different particles size ranges from the gas atomization process. Backscattered electron images indicate the different morphologies from spherical-like to plate-like shapes depending on the particle size range.

The weight percentages for different particle size ranges of gas atomized powders are presented in Figure 6. The gas atomization process produced ~35 wt.% of the powder of interest, i.e., inferior to 45 µm, which presented spherical or near-spherical morphology essential to ensure good fluidity upon HVOF spraying deposition. Analyzing the SEM images for particle size ranges from <45 µm to >250 µm, it is observed that the morphology changes from near-spherical to plate-like splats. Besides the detrimental effect of the plate-like morphology during feeding of powder in the HVOF gun, particles superior to 45 µm are also tricky to completely melt during combustion resulting in low adhesion and compaction of the droplets. The spray forming was also used as an alternative technique to process the higher GFA alloy (63.5Fe25Cr7Ni4.5B) because of its characteristics to produce a material with high levels of homogeneity directly from the liquid, even with boron additions [33]. Additionally, it can be mentioned that spray forming can produce bulk materials in the form of tubes or billets, in other words, this processing technique is closer to a potential application when compared to the melt-spinning ribbons and copper-mold casting.

**Figure 7:**
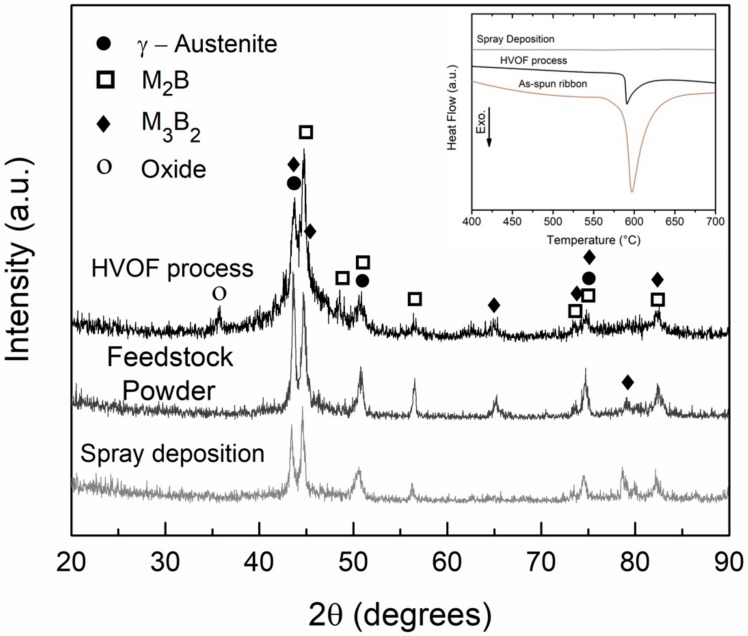
XRD curves of 63.5Fe25Cr7Ni4.5B spray deposited samples and feedstock powders and resulting HVOF coatings. Inset: DSC curves for 63.5Fe25Cr7Ni4.5B samples produced by melt-spinning, HVOF process, and spray deposition.

The XRD curves of the feedstock powders and the coatings produced by HVOF are exhibited in Figure 7, together with the sprayed deposit sample diffractogram. The sprayed deposit is completely crystalline as indicated by the well-resolved peaks. Differently, the HVOF coating presents a broad halo with crystalline peaks, comparable to that displayed by the feedstock powder. The DSC curves, inset in Figure 7, reinforces the XRD results regarding the formation of amorphous phase. Compared with the as-spun ribbons which can be considered as fully amorphous, the amorphous phase content of the samples can be estimated through the following equation: V_f_ = ΔH_sample_/ΔH_ribbon_, where ΔH_sample_ is the crystallization enthalpy of the HVOF coatings or sprayed deposits and ΔH_ribbon_ is the crystallization enthalpy of the as-spun ribbons. Even though the morphologies of the ~10 mg analyzed samples in DSC were different (cut ribbons, peeled coatings, and piece of deposit), the obtained curves can be used for semi-quantitative calculations of the amorphous content. As a result, the estimated amorphous fraction of the HVOF coatings is only about 20%, and that of the sprayed deposit was 0% since no significant exothermic event around 595 °C related to the crystallization was observed in the DSC curve (see inset Figure 7). We shall come back to this point in the discussion of the corrosion behavior of the samples, Section 3.3.

Gas atomization to produce feedstock powders, and spray deposition, are similar regarding the first steps, i.e., in both processes the alloy is molten without excessive overheating and then sprayed using high-pressure inert gas ensuring low oxidation. Indeed, any peak attributed to oxide phases was not observed in the feedstock powder and sprayed deposits XRD patterns, Figure 7. Contrary, a pronounced peak related to iron-oxide is observed for the HVOF coating pattern. An intrinsic variation in the size and thermal history exist for each individual particle exposed to the HVOF flame during spraying deposition. The smaller particles are more prone to undergoing overheating and their exposition to the atmosphere between the HVOF gun and the substrate induce oxidation which explains the non-negligible presence of iron oxide.

**Figure 8:**
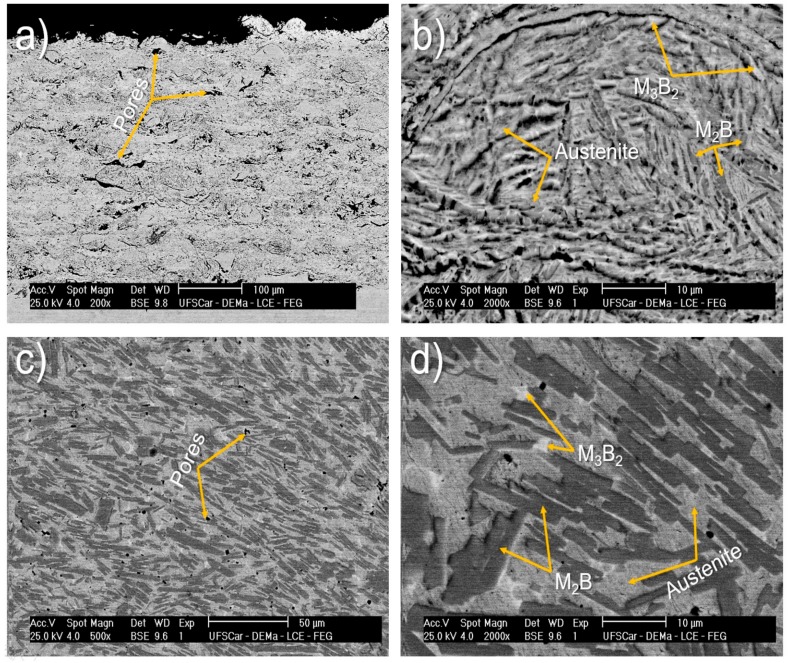
SEM backscattered electron image of 63.5Fe25Cr7Ni4.5B (**a**,**b**) HVOF coatings and (**c**,**d**) deposit produced by spray deposition. Phases were identified based on local EDS analyses and the thermodynamic calculation results. As the M_3_B_2_ borides are rich in Mo they appear as bright phases in the images. M_2_B borides appear as dark phases since M = Fe,Cr.

Different microstructures are observed in Figure 8 for spray deposited and HVOF coating samples. The HVOF coating exhibits a porous layer of 370 ± 20 µm in thickness (Figure 8a) while the sample processed by spray deposition presents a microstructure composed of rounded porosity (Figure 8c) with high amounts of (Fe,Cr)_2_B borides more refined than those presented in Figure 5 when cast in a copper mold. From Figure 8d, it is also possible to observe the presence of the eutectic borides (white regions) in an austenitic matrix. This microstructure corresponds well to what was predicted in the thermodynamic calculations (see Figure 4) since the spray forming is not considered a rapid solidification processing technique due to its last solidification stage, in which the cooling rates are considerably lower than at the atomization stage. The HVOF has a higher solidification rate than spray deposition and thus it is more prone to induce amorphous phase formation. Even though the cooling rates imposed by the HVOF process upon deposition were not sufficiently high to completely suppress the crystallization, the resulting microstructure is refined (see Figure 8b) especially when compared to that of sprayed deposits, Figure 8d. In fact, these processes have a quite different microstructure evolution, leading to different microstructures.

### 3.3. Corrosion Behavior

**Figure 9:**
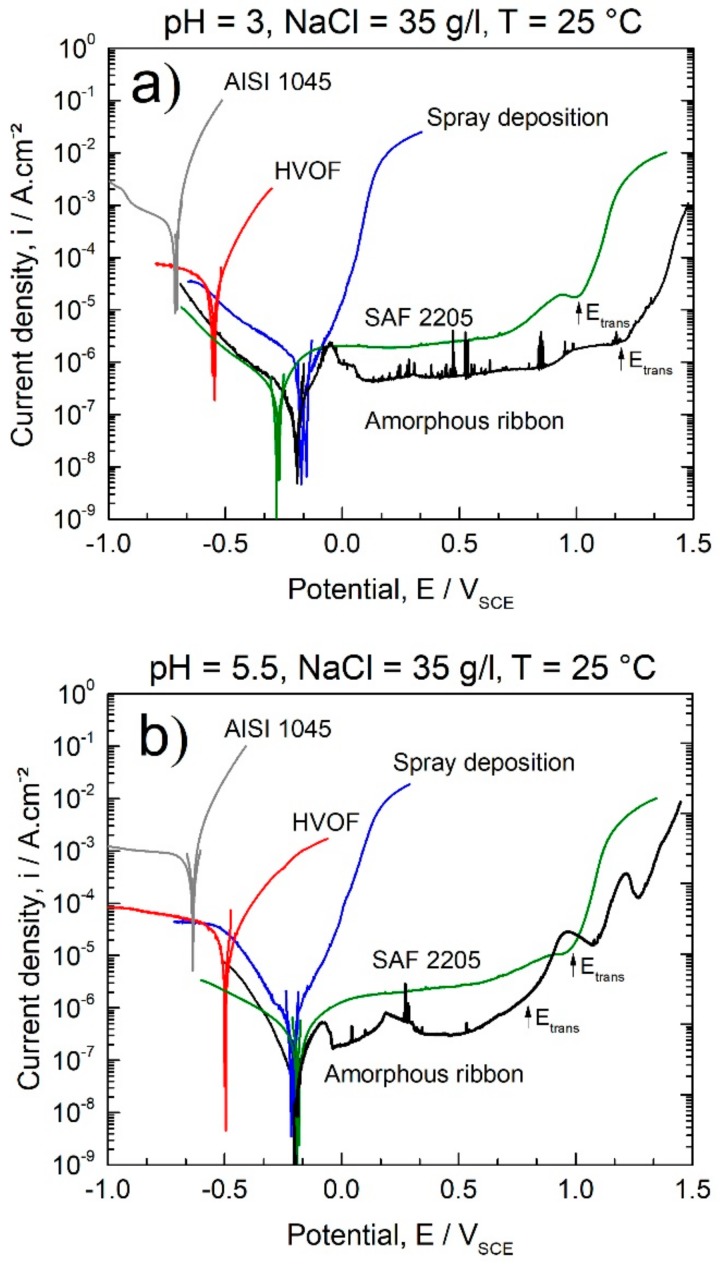

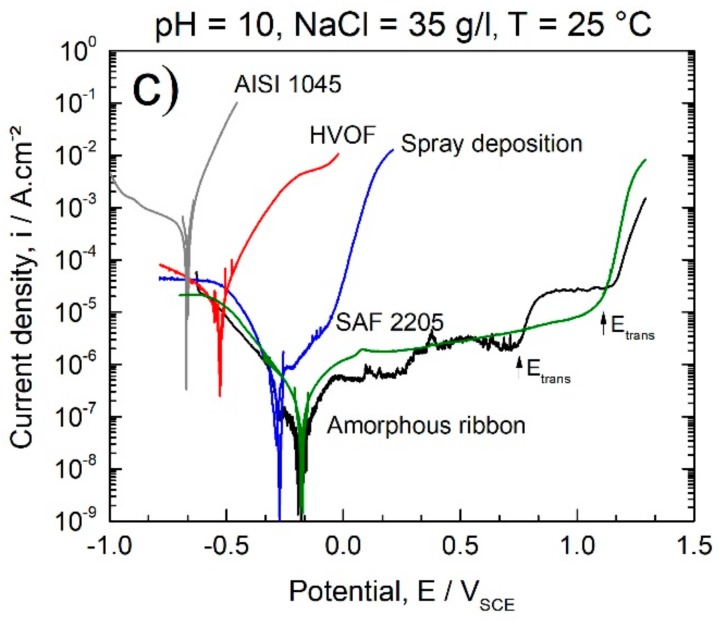
Polarization curves of 63.5Fe25Cr7Ni4.5B samples from melt-spinning, spray deposition, and the HVOF process analyzed in chloride-rich electrolyte, 35 g/L, at different pH: (**a**) pH = 3.0, (**b**) pH = 5.5, and (**c**) pH = 10.0. Results of AISI 1045 steel and of SAF 2205 stainless steel added for comparison purposes. E_trans_ stands for transpassivation potential.

To evaluate the corrosion behavior of amorphous and crystalline 63.5Fe25Cr7Ni4.5B samples produced through different routes (melt-spinning, spray deposition, and HVOF processes), potentiodynamic polarizations in chloride-rich media at different pH were carried out (Figure 9). The ensemble of the electrochemical parameters obtained from potentiodynamic polarization curves is summarized in Table 2. Three different electrochemical behaviors can be observed, regardless of the pH value. Fully amorphous ribbons present lower *i_corr_* and nobler *E_corr_* than crystalline samples obtained by HVOF and spray deposition processes. Since all the samples have the same chemical composition, the high corrosion resistance of amorphous ribbons may be attributed to the absence of crystallographic defects, uniformity of the passive film, as well as the intrinsic higher activity of amorphous surfaces. Indeed, the high reactivity of the amorphous alloy surface favors the rapid dissolution of low corrosion resistant components such as iron and the formation of uniform passive films enriched in passivating elements such as Cr, Ni, and Mo. Thus, a broad passivation plateau was observed upon anodic polarization, acting as an effective barrier against anodic dissolution assuring negligible corrosion rates. Amorphous ribbons present corrosion resistance even superior to the highly resistant SAF 2205 super duplex stainless steel as observed by the inferior *i_corr_* values and lower current density at the passivation plateau (Table 2 and Figure 9). Besides having superior Cr and Ni content compared to the SAF 2205 SS, the amorphous structure of the samples produced by melt-spinning is also responsible for enabling the outstanding corrosion performance in alkaline and acidic chloride rich media. The transpassivation potential, E_trans_, of the amorphous ribbons in acid chloride-rich medium is slightly superior than that of the SAF 2205 SS, Figure 9, but inferior at pH 5.5 and 10. Some metastable pitting characterized by fast-current spikes in pH 5.5 and more pronounced in pH 3.0 were also observed along the anodic polarization of the amorphous ribbons, but no stable pitting was induced even for polarizations as large as +0.6 V.

Samples produced by spray deposition exhibited *i_corr_* of about one order of magnitude superior to the one for amorphous as-spun ribbons at pH = 10.0 and 5.5. In acid media, pH 3, the differences between the *i_corr_* values of spray deposited and fully amorphous samples were smaller than at pH 5.5 and 10. Compared with fully amorphous samples, specimens produced by spray deposition showed higher susceptibility to corrode upon anodic polarization, as can be observed by the monotonic increase in the current density with the potential. Defects such as grain, grain boundaries, porosity, second-phase, and even dislocations induce the formation of galvanic couples, being preferential sites for the onset of corrosion. Additionally, the formation of chromium and molybdenium-rich borides, such as (Fe,Cr)_2_B and (Fe,Mo)_3_B_2_, decrease the content of corrosion resistant alloying elements in the matrix compared to the fully amorphous structure and, therefore, decrease the corrosion resistance [34].

HVOF samples presented lower *E_corr_* and higher *i_corr_* (up to 3 orders of magnitude) than the as-spun amorphous ribbons. The behavior of the HVOF coatings in different media is similar regardless of the pH, i.e., polarization curves characterized by the rapid increase of the anodic current density with the increase of the potential. In addition to the deleterious crystalline phases that deplete the matrix in Cr and promotes galvanic coupling [35], porosity significantly impairs the corrosion behavior of coatings [36,37]. Porosity acts as channels for the entrance of aggressive media, inducing differential aeration zones, also representing potential zones for the initiation of pitting corrosion. The HVOF process generally produces more porous coatings compared to spray deposition (see Figure 8) which explains the lowest corrosion resistance of HVOF coatings observed in this study.

The general trends for corrosion behavior indicate that the *E_corr_* are nobler and *i_corr_* lower in the following sequence: Amorphous ribbons, deposits obtained by spray forming, and HVOF coatings. The 63.5Fe25Cr7Ni4.5B amorphous alloy is highly resistant to corrosion (*i_corr_* of about 10^−8^ A/cm²) in chloride-rich acidic and alkaline environments with corrosion resistance even superior than that of the highly resistant SAF 2205 base alloy. Compared with the fully amorphous samples, deposits (fully crystalline) and coatings (~20% of amorphous phase) of the same composition obtained, respectively, by spray deposition and the HVOF process exhibited lower corrosion resistance in chloride-rich media due to the crystalline defects, as well as inherent porosity from the processing route. From these results, it could be highlighted the outstanding beneficial effect of the amorphous structure on the improvement of the corrosion resistance of stainless alloys steel. Although being less corrosion-resistant than the amorphous alloys, the HVOF and spray deposited specimens exhibit higher corrosion resistance than the substrate AISI 1045 (see Table 2 and Figure 9), with the advantage of being reinforced with borides reported to be extremely hard and resistant against wear [38], Figure 8.

## 4. Conclusions

- The GFA of boron-modified duplex stainless steel, 68-xFe25Cr7NixB (x = 3.5, 4.5, and 5.5 wt.%) was investigated. The 63.5Fe25Cr7Ni4.5B alloy composition presented the higher GFA, and fully amorphous ribbons were obtained by melt-spinning.
- The thermodynamic calculations predicted the stable phases, and from this result, it was possible to understand the microstructure evolution of the copper mold cast alloys for different cooling rates. These results were also of importance to understand the formed microstructure from the HVOF and spray deposition processes.
- HVOF and spray depositions were performed in the alloy with higher glass forming ability (63.5Fe25Cr7Ni4.5B). In both cases, the cooling rates were not high enough to ensure fully amorphization. The crystalline phases formed were primary (Fe,Cr)_2_B and eutectic (Fe,Mo)_3_B_2_ borides in an austenitic matrix. The microstructure of the HVOF coating was more refined than that of the sprayed deposits, however, with higher levels of porosity.
- Fully amorphous 63.5Fe25Cr7Ni4.5B ribbons exhibited excellent corrosion resistance in chloride-rich alkaline and acid media with *i_corr_* of about 10^−8^ A/cm^2^ and broad passivation plateau.
- Coatings and deposits of the same composition as for the ribbons (63.5Fe25Cr7Ni4.5B) produced by the HVOF process and spray deposition, respectively, exhibited lower corrosion resistance due to the presence of porosity and crystalline defects. However, their corrosion resistance was higher than that of the AISI 1045 steel used as substrate, with the advantage of being reinforced with hard borides, resulting in a composite microstructure promising to be resistant against wear.

## Figures and Tables

**Table 1:** The 68-xFe25Cr7NixB (x = 3.5, 4.5, and 5.5 wt.%) real alloy-compositions used for thermodynamic calculations. Composition of the base alloys, SAF 2205, and of the substrate, AISI 1045, for high-velocity oxygen fuel (HVOF) and spray deposition also included.

Alloy/wt.%	C	Si	Mn	Cr	Ni	Mo	S	N	P	B	Fe
64.5Fe25Cr7Ni3.5B	0.08	0.35	1.02	25	7	1.67	<0.01	0.08	0.009	3.5	bal.
63.5Fe25Cr7Ni4.5B	0.09	0.35	0.82	25	7	1.47	<0.01	0.07	0.007	4.5	bal.
62.5Fe25Cr7Ni5.5B	0.11	0.36	0.78	25	7	1.29	<0.01	0.06	0.006	5.5	bal.
SAF 2205	0.02	0.35	1.57	22.60	5.38	2.58	0.01	0.13	0.010	-	bal.
AISI 1045	0.47	0.15	0.63	0.055	0.04	0.02	0.02	-	0.014	-	bal.

**Table 2:** Corrosion properties of 63.5Fe25Cr7Ni4.5B samples obtained from potentiodynamic polarization curves for amorphous ribbons, coatings produced HVOF process and deposits from spray forming. Results of AISI 1045 steel and SAF 2205 added for comparison reasons. Potentials measured against the saturated calomel electrode (SCE) reference.

Samples	Media: pH = 10.0		Media: pH = 5.5		Media: pH = 3.0	
	*E_corr_* (mV)	*i_corr_* (µA/cm^2^)	*E_corr_* (mV)	*i_corr_* (µA/cm^2^)	*E_corr_* (mV)	*i_corr_* (µA/cm^2^)
HVOF coating	−530 ± 10	30 ± 5	−480 ± 3	30 ± 6	−530 ± 5	20 ± 9
Spray deposition	−310 ± 30	0.52 ± 0.04	−230 ± 20	0.7 ± 0.1	−180 ± 30	2.0 ± 0. 8
Amorphous ribbon	−120 ± 25	0.05 ± 0.01	−200 ± 10	0.07 ± 0.02	−190 ± 20	0.16 ± 0.03
SAF 2205	−183 ± 21	0.2 ± 0.1	−181 ± 36	0.3 ± 0.2	−281 ± 12	0.3 ± 0.1
AISI 1045	−666 ± 15	500 ± 80	−632 ± 20	800 ± 100	−715 ± 11	400 ± 20
